# Anesthésie pour prothése totale de la hanche: à propos de 50 cas

**DOI:** 10.11604/pamj.2015.22.379.6938

**Published:** 2015-12-17

**Authors:** Issam Serghini, Youssef Qamouss, Mohamed Zoubir, Jaafar Salim Lalaoui, Idrissi Khalid Koulali, Mohamed Boughalem

**Affiliations:** 1 Pôle Anesthésie- Réanimation, Hôpital Militaire Avicenne, Faculté de Médecine et de Pharmacie, Université Cadi Ayyad, 40010 Marrakech, Maroc; 2 Pôle de Traumatologie et Orthopédie, Hôpital Militaire Avicenne, Faculté de Médecine et de Pharmacie, Université Cadi Ayyad, 40010 Marrakech, Maroc

**Keywords:** Arthroplastie totale de la hanche, anesthésie, évaluation préopératoire, complications per et post opératoires, Total hip arthroplasty, anesthesia, preoperative evaluation, surgery complications

## Abstract

La chirurgie de la prothèse totale de la hanche (PTH) est une chirurgie fonctionnelle qui consiste en un remplacement d'une articulation endommagée afin d'améliorer la qualité de vie du patient. L'anesthésie pour PTH exige une préparation rigoureuse à l'intervention, la consultation d'anesthésie sera donc la clef de cette réussite. Nous avons réalisé une étude rétrospective concernant 60 arthroplasties totales de la hanche implantées chez 50 patients adultes, colligée au sevice de Traumatologie et de chirurgie orthopédique à l'Hôpital Militaire Avicenne de Marrakech sur une période étalée de Janvier 2010 au Décembre 2012. Nous avons évalué la prise en charge anesthésique: pré, per et postopératoire des patients opérés pour une PTH. La moyenne d’âge était de 56,5 ans, le sex-ratio était de 1,63 en faveur des hommes. L'indication prédominante était la coxarthrose primitive. L'anesthésie générale était la technique anesthésique la plus utilisée (66% des cas), l'intubation difficile était rencontrée chez 6% de nos patients. La durée moyenne de l'acte chirurgical était de 114 +/- 25,33 mn. 12% de nos patients ont présenté une hypotension artérielle peropératoire. L'incidence de la transfusion homologue perop était de 14%. Nous avons noté 08 cas de complications postop: 03 cas d'infection de la PTH 15 jours après l'intervention, 03 cas de descellement aseptique, 01 cas de luxation de PTH et 01 cas de descellement septique avec un recul moyen de 54 mois.

## Introduction

Le nombre de PTH posés par an est en perpétuelle augmentation dans les pays industrialisés, mais aussi au Maroc du fait de l'amélioration de la qualité de vie et de l'augmentation de l'espérance de vie. La chirurgie pour arthroplastie totale de la hanche est une intervention dont la mortalité est non négligeable ce qui implique une gestion rigoureuse des différents risques encourus: anesthésique, hémorragiques, infectieux et thromboemboliques. L'objectif de ce travail est d’évaluer la prise en charge anesthésique pré, per et postopératoire chez des patients opérés pour une PTH en insistant sur la place de la consultation pré-anesthésique, l'antibioprophylaxie, les besoins transfusionnels et techniques d’économie de sang, la tromboprophylaxie et l'analgésie postopératoire.

## Méthodes

Nous avons effectué une étude rétrospective sur une période de Janvier 2010 à Décembre 2012. Une série de 60 arthroplasties totales de la hanche chez 50 patients adultes a été répertoriée. Dans notre travail, nous avons inclus tous les patients adultes opérés pour PTH et exclus tous les patients opérés pour une chirurgie de la hanche et qui ont bénéficié d'une hémiprothèse ou une chirurgie non prothétique. Nous avons exploité les dossiers médicaux d'hospitalisation et les registres du bloc opératoire du service de Traumatologie et du service de réanimation polyvalente à l'Hôpital Militaire Avicenne de Marrakech. Nous avons procédé à une recherche bibliographique au moyen de Medline, science direct et pubmed. L'exploitation des différents dossiers a été faite par une fiche d'exploitation comportant une période pré, per et postopératoire.

## Résultats

**Figure 1:**
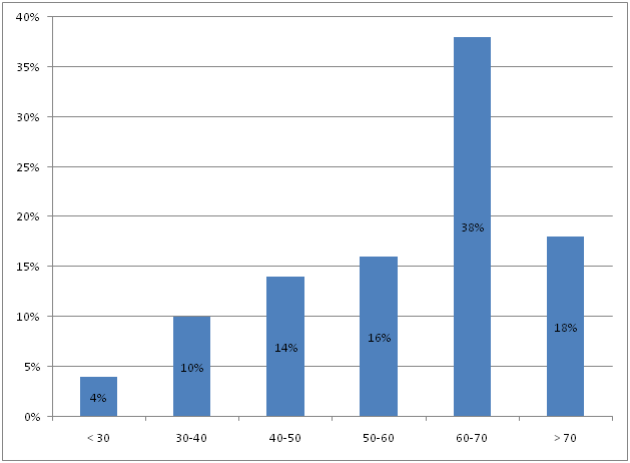
Répartition par tranche d’âge

**Répartition des cas en fonction de l’âge**: (Figure 1) la moyenne d’âge des patients était de 56,5 ans avec des extremes de 21 ans et 80 ans. La répartition par tranche d’âge montre un pic pour la tranche d’âge 60-70 ans.

**Répartition en fonction du sexe**: 62 des patients étaient de sexe masculin, tant dis que les femmes représentaient 38% soit un sexe ratio de 1,63.

**Répartition en fonction de l'IMC**: l'indice de masse corporelle moyen était de 28,55 Kg/m^2^: 28 patients avaient un surpoids, 15 ont été obèses et 07 patients avec un poids normal.

**Antécédents pathologiques**: ATCD médicaux; - Diabéte: 08; * 05 patients sous antidiabétiques oraux, * 03 patients étaient sous insuline intermédiaire, - HTA: 07; * 04 patients étaients sous inhibiteurs calcique IC, * 03 patients étaient sous inhibiteurs de l'enzyme de conversion IEC, -Cardiopathie: 04; * ischémique: 01, * insuffisance cardiaque: 03 patients sous: bétabloquant, inhibiteur calcique et diurétiques, -BPCO: 01 patient sous corticothérapie et bronchodilatateurs; -Maladie de Behcet: 01 patient; -Tuberculose: 04 patients (traités par le schéma 2RHZE/4RH); * pulmonaire: 03 patients, * extra-pulmonaire osseuse: 01 patient, - Insuffisance rénale chronique: 01 patient hémodialysé; - SPA: 08 patients étaient sous traitement médical (AINS et méthotrexate); - ATCD chirurgicaux; - 01 patient avait une luxation de la hanche traitée orthopédiquement; - 01 patient avait bénéficié auparavant d une biopsie au niveau de la hanche opérée pour suspicion de tuberculose osseuse; - 02 patients avaient une fracture du radius traitée chirurgicalement; - 01 patient avait une fracture de l humérus traitée orthopédiquement; - 01 patient avait une fracture bimalléolaire traitée chirurgicalement; - 04 patients avaient une fracture du col fémoral iopérés par vissage, vis plaque DHS ou par prothése céphalique; -Oculaire (cataracte): 01 seul cas.

**Figure 2:**
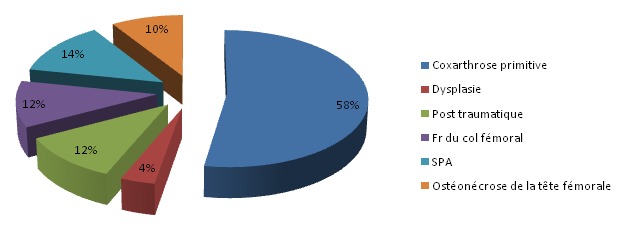
Répartition des cas selon le facteur étiologique

**Indications de la PTH**: (Figure 2) Coxarthrose; *Primitive: 29, *Dysplasie: 02, *Post-traumatique: 06, Fr du col femoral: 06; SPA: 07; Osteonecrose de la tête femorale: 05; Nous avons noté que plus de la moitié des indications de la PTH était des coxarthroses primitives.

**Bilan pré-opératoire**: Le bilan préopératoire a été orienté en fonction du contexte clinique. Tous nos patients ont bénéficié d un bilan paraclinique préopératoire de base comportant: Un bilan infectieux: NFS CRP Blondeau Panoramique dentaire avec consultation ORL. Bilan pré-anesthesique fait de: Groupage sanguin, bilan d hemostase TP TCK Bilan rénal: urée /créat, Glycémie à jeun, Radio de thorax et ECG. L’échographie cardiaque a été réalisée chez 02 patients. L'ECBU a révélé chez 02 patients une infection urinaire ayant nécessitée un traitement ATB.

**Figure 3:**
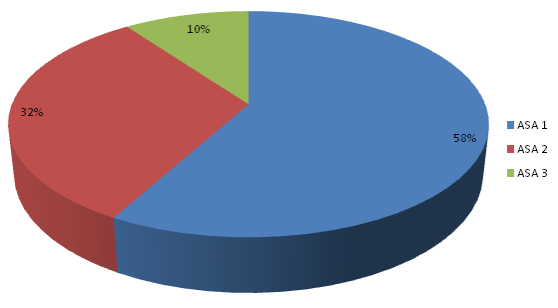
Répartition des patients selon la classification ASA

**Répartition des patients selon la classification ASA**: (Figure 3) ASA 1: 29 patients soit 58%. ASA 2: 16 patients soit 32%. ASA 3: 5 patients soit 10%. ASA 4: aucun patient.

**Préparation et installation du malade**: tous nos patients ont bénéficié d'une douche la veille de l'opération et d'une préparation locale qui a consisté à un rasage du membre inférieur et du pubis avec une désinfection cutanée de la région opératoire par de la Bétadine dermique avant l'intervention. L'intervention s'est déroulée dans une salle réservée exclusivement à la chirurgie aseptique. Tous nos patients ont été installés en décubitus latéral selon le côté opéré sur une table orthopédique. Avec 2 appuis antérieurs pubien et thoracique, et 2 appuis postérieurs sacré et thoracique. Un drap plié en deux ou un sac spécial est disposé verticalement, le long de la table, du côté verticale du patient, de façon à pouvoir y glisser le membre inférieur au cours du temps fémoral.

**Figure 4:**
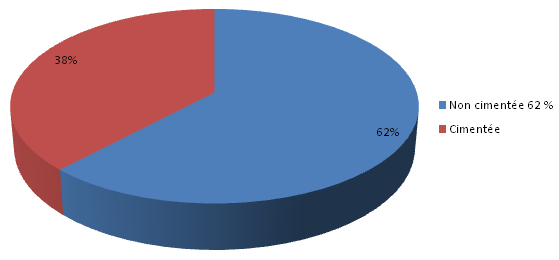
Répartition en fonction du matériel utilisé

**Figure 5:**
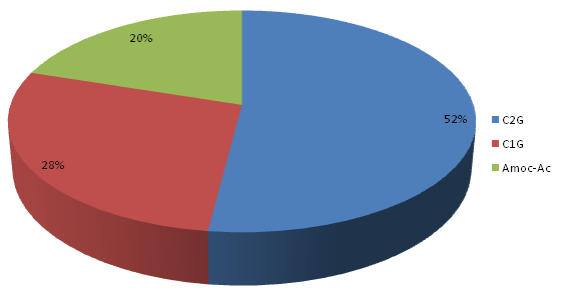
Répartition en fonction du type d'antibiotique

**Matériel utilisé et antibioprophylaxie**: (Figure 4, Figure 5) toutes les PTH mises en place avaient un couple de frottement métal polyéthylène type métabloc. Nous avons utilisé la PTH cimentée chez 23 cas soit 38,3%, tandis que la PTH non cimentée a été utilisée chez 37 cas soit 61,7%. La céphalosporine de 2 éme génération était utilisée chez 26 patients soit 52%. La céphalosporine de 1 ére génération était utilisée chez 14 patients soit 28%. L'amoxicilline protégée était utilisée chez 10 patients, soit 20%.

**Technique d'anesthésie et durée d'intervention**: technique anesthésique: dans notre série, l'anesthésie utilisée varie entre une anesthésie générale et une rachianesthésie avec une préférence de l'anesthésie générale. * 17 patients ont été opérés sous rachianesthésie. La rachianesthésie a été assurée par la Bupivacaine hyperbare associé à des faibles doses de morphiniques liposolubles fentanyl. *33 patients ont été opérés sous AG: - induction: Narcose: Diprivan: 28 patients; Hypnomidate: 05 patients. Analgésie: Fentanyl: 29 patients; Sufentanyl: 04 patients. Curarisation: Norcuronium: 23 patients; Rocuronium: 06 patients; Cisatracurium: 04 patients. Entretien: l'entretien de l'anesthésie a été assuré par: l'halothane, l'oxygène et le dioxyde d'azote chez tous les patients. Réveil: tous nos patients ont bénéficié d'une analgésie postopératoire multimodale débutée 30 minutes avant la fin de l'intervention chirurgicale: paracétamol, AINS et Néfopam. Tous nos patients ont reçu une réinjection de morphiniques sauf 2 patients qui ont bénéficié d'une analgésie péridurale. Intubation difficile: L'intubation était facile chez 30 patients L'intubation était difficile chez 03 patients: 02 patients avaient une SPA et 01 patient sans cause évidente. Durée d'intervention chirurgicale: La durée moyenne des interventions chirurgicales était de 114 +/- 25,33, avec des extrêmes allant de 70 mn à 180 mn.

**Les pertes sanguines**: on rappelle que l'estimation des pertes sanguines est la somme du saignement recueillis à travers l'aspiration du champ opératoire et le sang recueilli dans les compresses. La moyenne du saignement en peropératoire était de 750 ml avec un minimum de 150 ml et un maximum de 1300 ml. Parmis les 50 patients étudiés, 7 ont nécessité une transfusion homologue peropératoire, soit une incidence de 14%. Aucun cas de complications transfusionnelles immédiates n'a été rapporté parmi les patients transfusés.

**Résultats de la surveillance per-opératoire**: Dans notre série, le monitorage peropératoire a été assuré pour tous nos patients comprenant: un electrocardioscope, un dynamap, un capnographe, un oxymètre de pouls, un curarométre et Hemocue sur sang artériel. La FC, PA ont été notées toutes les 5 mn depuis le début de l'intervention jusqu′à 15 mn, puis toutes les 15 mn jusqu’à une heure, ensuite toutes les demi-heures jusqu’à la fin de l'intervention. Aucune modification significative de la FC n'a été notée. La baisse de la PA a été plus importante à la 15 éme min. le nombre de patients ayant présenté une hypotension artérielle était de 6 soit 12%. Le recours aux sympathomimétique était nécessaire chez 4 patients, avec faibles doses d’éphédrine 6 mg +/- 3 mg chez 3 patients, un seul patient a nécessité de fortes doses 15 mg.

**Figure 6:**
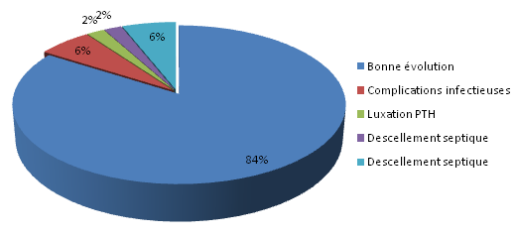
Répartition des cas selon la présence ou non de complications

**Figure 7:**
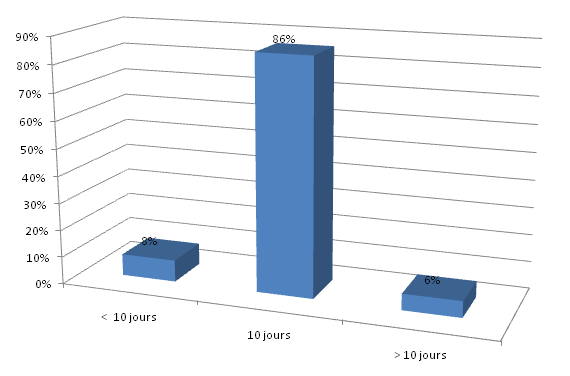
Répartition des cas selon le séjour hospitalie

**Résultats de la surveillance postopératoire**: (Figure 6) complications infectieuses: dans notre série, 03 patients (6%) ont présenté une infection sur la prothèse 15 jours après le geste opératoire et qui ont bénéficié d'une antibiothérapie adaptée à l'antibiogramme avec un drainage. Complications thromboemboliques: dans notre série, nous n'avons noté aucun cas de thrombophlébite, ni d'embolie pulmonaire. Luxation de prothése: dans notre série, nous avons noté un seul cas de luxation de PTH nécessistant une réduction sous AG avec traction collée. Descellement septique: un de nos patients a présenté un descellemnt septique de PTH apré 5 ans de recul. Descellement aseptique: dans notre série, 3 de nos patients ont présenté un descellement de PTH unipolaire du composant cotyloidien après 5 ans de recul, et deux entre eux ont bénéficié d'une reprise de PTH. Le recul postopératoire: tous les patients ont été revus. Le recul moyen était de 54 mois (4 ans e demi). Le séjour hospitalier: le séjour hospitalier moyen était de 10 jours avec des extrêmes de 08 et de 15 jours (Figure 7). **Décès**: Nous n'avons noté aucun décès, un seul patient a développé un sepsis 5 ans après la mise en place de la prothèse, et qui a bien évolué sous traitement par la suite.

## Discussion

L’âge comme élément épidémiologique est important à prendre en considération dans la pose d'une PTH. C'est un facteur important déterminat le résultat fonctionnel et la longévité de la prothése, avec un meilleur résultat entre 45 et 75 ans [1]. La moyenne d’âge dans notre série est de 56,5 ans, cette moyenne d’âge basse est expliquée par le jeune âge de la population marocaine par rapport à la population occidentale et par la fréquence des pathologies touchant le sujet jeune: coxites inflammatoires, coxarthrose post traumatique. Classiquement, il existe une prédominance féminine parmi les patients candidats à une PTH [2, 3]. Dans notre série, nous avons noté une prédominance masculine. On trouve également cette prédominance masculine dans la série de Glas de PTH pour coxarthrose post-traumatique [4]. Dans notre série, cette prédominance peut être expliquée par la fréquence des indications post-traumatiques de la PTH d'une part, et d'autre part par la fréquence de la coxarthrose chez l'homme en rapport avec des particularités de l'hôpital (hôpital militaire). Les indications de la PTH restents dominées par la coxarthrose [5], la coxarthrose primitive constitue la première indication de la PTH dans notre série (58%), rejoignant les données de la littérature dans la série de Charnley [2], 70% des prothèses sont implantées pour une coxarthrose primitive, ce pourcentage rejoint celui de Callaghan [6] (72%), de Timo [7] (78%). La consultation pour chirurgie prothétique, est idéalement programmée 1 mois avant l'intervention. Elle s'oriente autour de l’évaluation cardio-respiratoire et rénale, évaluation des problémes de coagulation, de la recherche d'un foyer infectieux, et de la mise en place d'une stratégie pré, per et post opératoire tenant compte de la pathologie du patient, de la chirurgie proposée et des possibilités d'autotransfusion; Dans notre étude ce délai moyen de consultation pré-anesthésique n'a pas été respecté. Vue l'interaction d'un certain nombre de médicaments, pris en préopératoire par les patients, soit avec l'anesthésie, soit avec la chirurgie elle-même, certains seront maintenus, dautres seront soit arrêtés soit substitués. Pour les antiagrégants plaquettaire (AAP), la problématique reste la même: quel est le risque thrombotique cardiovasculaire à l'arrêt de ces produits? Quel est le risque hémorragique si on ne les arrête pas. La littérature à propos de l'acide acéthylsalicylique était déjà pauvre et ne concernat en grande partie que la chirurgie cardiaque [8]. Le flurbiproféne (Cébutid), à la dose de 50 mg deux fois par jour, est le seul AINS à avoir une autorisation de mise en marché (AMM) en France pour son effet sur les plaquettes. Son indication comme relais de l'aspirine ou des thiénopyridines n'est pas validé. Les HBPM ont fait leur preuve de leur efficacité dans l'angor instable mais en association avec l'aspirine [9]. Néanmoins certaines équipes de cardiologie préconisent le remplacement du traitement antiplaquettaire par une HBPM à dose curative, éventuellemnt associé au Flurbiproféne. L'attitude adopter vis-à-vis des nouveaux AAP est la même que celle vis-àvis de l'aspirine, que ce soit en urgence ou non [10]: en cas de risque hemorragique, il faut substituer ces agents, retarder ou modifier le geste chirurgical, transfuser les plaquettes; en cas de risque thrombotique, il convient de réduire le délai d'arrêt de ces agents jusqu’à 3 jours si le taux de plaquettes initial est correct.

La gestion péri-opératoire d'un patient traité par anticoagulant ne se résume plus à la siple prescription d'un arrêt du traitement associé si besoin à un relais. Le risque lié à la thrombose peut avoir des répercussions cliniques plus délétèrent que le risque hémorragique. Cette prise de conscience a permis de mieux cerner la conduite à tenir qui dépend du degré d'urgence de l'acte, du terrain thrombotique du patient et du risque hémorragique de la chirurgie et de l'anesthésie [11]. Dans notre expérience La prise d'AINS a été arrêtée 10 jours avant l'intervention chirurgicale dans la moitié des cas, et 5 jours avant pour le reste. L'arrêt 3 à 4 jours de l'AVK était chez 02 patients, et sont relayés par de l'héparine de bas poids moléculaire. 07 patients étaient sous traitements anti hypertenseur: L'IEC est les diurétiques ont été arrêtés 48 h avant l'intervention, les inhibiteurs calciques et les béta-bloquants ont été maintenus. 05 patients ont été sous antidiabétiques oraux, notre conduite à tenir: la programmation matinale de l'acte opératoire, arrêt de biguanides 48 h en préopératoire, arrêt de sulfamides la veille de l'opération puis relais par de l'insuline. Actuellement, il n'existe pas de différence entre AG et ALR médullaire au niveau de la morbidité et de la mortalité [12]. La consultation est le meilleur moment pour discuter avec le patient les avantages et les inconvénients des deux méthodes. La douleur postopératoire ne doit plus être considérérée comme un tribut obligatoire de la chirurgie osseuse des moyens d'analgésie efficace sont à la disposition des patients. Durant la période préopératoire, 40 à 80% des malades sont anxieux [13], l'objectif de prémédication est de permettre au patient durant la période préopératoire d’être sédaté et exempt de toute angoisse en étant parfaitement stimulable et coopératif. Dans notre série, l'Hydroxyzine a été utilisée en prémédication par voie orale (1,5 à 2 mg/kg). Il s'agit d'une chirurgie dite ‹propre›de classe I d'altemeir. Le risque infectieux spontanée est de 3à 5% est ramené à 0,4% si l'antibioprophylaxie est bien menée. La cible est le staphylocoque doré métisensible (SMS), trouvé dans plus de 70% des infections, mais aussi le streptocoque et la colo bacille [14]. L'antibiotique doit être injecté par voie intraveineuse au moment de l'induction anesth ésique à la dose préconisée. Une ou plusieurs injections de demi-doses doivent être effectuées toutes les demi-vies de l'antibiotique si l'intervention n'est pas terminée. Dans notre série l'antibioprophylaxie a été utilisée chez tous les patients, elle était à base d'une céphalosporine de deuxième ou de première génération, l'amoxicilline protégée a été utilisée chez 10 patients soit 20%. Cette antibioprophylaxie a été entretenue 48 heures en postopératoire. Malgré cette antibioprophylaxie nous avons noté 03 cas d'infection postopératoire précoce d'origine nosocomiale, qui ont bien évolué sous traitement antibiotique adapté avec drainage. Nous rapportons aussi un seul cas de descellement septique (2%) ayant necessité une antibiothérapie générale avec dépose de sa prothèse, et mise en place de ciment associé aux antibiotiques locaux, puis repose de la PTH après amélioration de l’état infectieux. Les problémes d'intubation difficile sont souvents rencontrés chez les patients atteints de polyarthrite rhumatoide avec subluxation atloido-axoidienne, ou ceux atteints de spondylarthrite ankylosante avec raideur du rachis cervical [14].

Actuellement, bien qu'il soit admis que la rachianesthésie réduit le temps opératoire et nécessite moins le recours à la transfusion [15], il est difficile de fixer une règle générale en donnant la préférence à un type d'anesthésie, générale ou locorégionale. Dans notre série, 66% étaient opérés sous AG et 34% sous rachianesthésie, dans une étude réalisée en 2009 par Mouilhadea [16], 89,3% des patients étaient opérés sous AG tant dis que 10,7% sous RA. On retient doc que le choix d'une technique anesthésique dépend de certains facteurs qui seront déterminants: les antécédents du patient, les habitudes du médecin anesthésiste, les conditions opératoires (durée, température de la salle, position.) et surtout, les préférences du patient bien informé dès la consultation d'anesthésie. Commun à toute anesthésie, le monitorage permet de surveiller l’état cardiorespiratoire du patient durant l'intervention. Il doit être en accord avec les recommandations de la SFAR [17]. Le sondage urinaire doit être d'indication large, du fait de l'immobilisation et de la retention urinaire post anesthésique. Dans notre série, le monitorage a été assuré pour tous les patients comprenant: un électrocardioscope, un oxymètre du pouls, un dynamap, un capnographe, un curamètre et un hémoglobunomètre sur sang artériel. L'intervention dure en moyenne deux heures, éviter l'hypothermie permet surtout de diminuer les frissons postopératoires, mai également susceptible de diminuer le saignement [18] et le risque infectieux [19]. Il semble actuellement que seules les couvertures à air chaud pulsé aient fait la preuve de leur réelle efficacité. L'utilisation du ciment (methacrylate de méthyle) expose le patient à des complicationsmortelles: arrêt cardiaque, collapsus et hypoxémie) [20]. Le tableau clinique est celui d'une défaillance cardiaque droite puis globale par dilatation droite avec compression des cavités gauches [21]. Le traitement est symptomatique: oxygénation, catécholamines et remplissage vasculaire. En 1999, Bierbaum, dans une étude à travers de 330 centres hospitaliers orthopédiques avec un effectif de 9482 patients, 57% ont bénéficié d'une transfusion, dont 66% de transfusion autologue et 34% homologue [22]. Alors que Borghi, l'incidence de transfusion homologue dans son étude ne dépassait paq 9,6% [23]. Dans notre étude, l'incidence de la transfusion sanguine est de 14% ce qui peut être expliqué par l'utilisation seule de la transfusion homologue, et par l’âge relativement jeune des patients. Salido démontre que le sexe féminin, le surpoids et la taille avaient un impact significatif sur le taux transfusionnel, ce taux n’était pas influencé par l’âge des patients [24]. Dans notre analyse des facteurs épidémiologiques prédictifs de la transfusion dans l'arthroplastie totale de la hanche, nous avons noté que plus l’âge n'est élevé, plus le besoin transfusionnel augmente. Chorois avait rapporté que les pertes sanguines pour athroplastie sont plus importantes quand la chirurgie est exécutée pour une coxarthrose destructive que pour une coxarthrose habituelle [25, 26]. Nous n'avons pas constaté de relation significative entre le recours à la transfusion et l’étiologie de la PTH. Dans une étude parue en Octobre 2006, Mauermann compare 330 patients ous AG et 348 patients sous rachianesthésie et démontre que la rachianesthésie réduit le temps opératoire de 7,1 mn et réduit les pertes sanguines de 275 ml. De ce fait, les patients opérés sous rachianesthésie nécessiteront moins le recours [15]. Nous avons souligné que parmi les 14% des patients transfusés, 71,5% étaient sous AG alors que 28,5% étaient sous RA. Nos résultats corroborent avec ceux de la littérature. Notre étude exclut la durée d'intervention chirurgicale comme facteur prédictif de la transfusion en chirurgie prothétique de la hanche. Nous avons tenu que les facteurs prédictifs à la transfusion peropératoire en termes de PTH sont l’âge et le type d'anesthésie.

La stase veineuse postopératoire est d'autant plus importante et durable que la douleur limite la mobilisation précoce. Mais il existe des facteurs biologiques associés. Certains sont généraux (thrombophilie constitutionnelle ou acquise). D'autres sont directement liés à l'acte opératoire (inflammation, facteur tissulaire.). Nous n'avons noté aucun cas de thrombophlébite, ni d'embolie pulmonaire. White [27] avait montré, dès 1998, à partir de données en réseau de l’état de Californie, que la médiane d'apparition des événements cliniques était de 17 jours après PTH. Dans leurs méta-analyses, Eikelboom [28] et Douketis [29] montraient qu'une prophylaxie prolongée réduisait le taux d'ETE symptomatique après PTH (1,4% vs 4,3%). Cela s'est traduit dans les recommandations nord-américaines et francaises. Les guidelines nord-américains ont légèrement évolué: 2004 [30], l'ACCP recommandait d’étendre jusqu’à 28-35 jours pour la PTH. Dans notre série, le traitement anticoagulant à base d'Enoxoparine a été poursuivi pour une durée de 43 jours, chez tous nos patients. L'arrivée de nouveaux anticoagulants oraux (NACO) a révolutioné la prise en charge du patient dans la prévention primaire des thromboses des PTH. Seul le Rivaroxaban (Xarelto) est commercialisée au Maroc. Cette chirurgie est très douloureuse. Le traitement de cette douleur permet, non seulement le confort du patient, mais aussi une mobilisation précoce, qui facilite la rééducaation et donc améliorer le pronostic fonctionnel. Dans notre étude, tous les patients ont bénificié d'une analgésie postopératoire multimodale débutée 30 minutes avant la fin de l'intervention chirurgicale par: le Paracétamol, les AINS et le Néfopam avec une réinjection de morphiniques sauf 02 patients qui ont bénéficié d'une analgésie péridurale. Les complications infectieuses surviennent dans les jours qui suivent l'opération et en général avant la fin de la convalescence habituelle [31]. Considérées comme une complication grave de la chirurgie prothétique, ces infections sont à un taux de 0,5% selon Duparc et peuvent aller jusqu’à 2% selon Garret. Ce taux a été fortement influencé par l'utilisation de l'antibioprophylaxie, la recherche et le traitement préopératoire de tout foyer infectieux chez le patient, le respect des règles d'hygiène et d'asepsie rigoureuse. Dans notre série nous avons noté 03 cas d'infection postopératoire précoce, qui ont bien évolué sous traitement antibiotique bien adapté et par des soins locaux avec drainage. Le descellement constitue la complication la plus fréquente des PTH [30]. Il peut être septique ou aseptique. Dans notre série nous avons eu 03 cas de descellemnt cotyloidien survenu à 5 ans de recul dont 202 cas ont bénéficié d'une reprise de PTH. Nous remarquons donc que notre pourcentage de descellemnt aseptique dans notre série (6%) rejoint celui de la littérature. Tandis que le descellemnt septique n'a été rapporté que chez 01 patient qui a bien évolué sous traitement. La luxation est, après le descellement, la deuxième complication susceptible de remettre en cause le résultat d'une arthroplastie totale de la hanche. Sa fréquence selon les séries publiées se situe entre 0,11 à 9%. Le traitement est d'abord orthopédique consistant en une réduction sous AG. Dans le cas d'une irréductibilité, d'interposition, de désassemblage prothétique, la réduction chirurgicale s'impose. Dans notre série, nous avons noté un seul cas de luxation de PTH ayant nécessié une réduction sous AG avec traction collée et dont l’évolution était favorable.

## Conclusion

L'arthroplastie totale de la hanche est devenue une pratique courante est bien codiié en chirurgie orthopédique. A travers l’étude de notre série, on se rend compte de la fréquence de plus en plus augmentée u nombre de PTH posées par an au Maroc, mais également des compétences nationales en matiére de la technique chirurgicale. Il est donc l'heure de mettre à l'existence un registre national marocain des PTH, qui va aider à standariser les attitudes, évaluer les résultats et tirer des conclusions ppour établir des consensus nationaux en matière de PTH.
